# Comparative Study of the Antioxidant Effects of Metformin, Glibenclamide, and Repaglinide in Alloxan-Induced Diabetic Rats

**DOI:** 10.1155/2016/1635361

**Published:** 2015-12-28

**Authors:** Bonaventure Chukwunonso Obi, Theophine Chinwuba Okoye, Victor Eshu Okpashi, Christiana Nonye Igwe, Edwin Olisah Alumanah

**Affiliations:** ^1^Department of Pharmacology and Toxicology, Faculty of Pharmaceutical Sciences, University of Nigeria, Nsukka 410001, Enugu State, Nigeria; ^2^Department of Biochemistry, University of Nigeria, Nsukka 410001, Enugu State, Nigeria; ^3^Synthetic Organic Chemistry Division, Department of Pure and Industrial Chemistry, University of Nigeria, Nsukka 410001, Enugu State, Nigeria

## Abstract

Diabetes mellitus is one of the serious global health problems affecting a significant proportion of both developed and developing countries. Overproduction of free radicals and oxidative stress has been associated with the development of diabetic complications. In the present study, the antioxidant effects of metformin (MET), glibenclamide (GLI), and repaglinide (REP) were evaluated in alloxan-induced diabetic rats. The findings from this study may possibly help in understanding the efficacy of these standard drugs in managing the complications arising from diabetes mellitus (DM). Alloxan (130 mg/kg BW) was administered as a single dose to induce diabetes. Four (4) groups of rats (*n* = 6) were used; group 1 served as diabetic control while groups 2, 3, and 4 were the diabetic test groups that received MET (25 mg/kg), GLI (2.5 mg/kg), and REP (0.5 mg/kg), respectively. The result of the study showed significant (*p* < 0.05) improvement in the altered antioxidant enzymes (SOD, CAT) and GSH concentration in diabetic treated rats compared with the diabetic control group. MET and REP produced significant effect on the MDA concentration while GLI showed insignificant reduction in the MDA concentration compared with the diabetic control. Findings from this study suggest that the administration of MET, GLI, and REP exerts significant antioxidant effects in alloxan-induced diabetic rats, thus contributing to the protective effect against oxidative stress-induced damage during diabetic complications.

## 1. Introduction

Diabetes is the most common metabolic disorder out of various lifestyle diseases associated with many complications such as diabetic ketoacidosis, hyperosmolar coma, cardiovascular problems, kidney failure, eye damage, nonketotic hyperosmolar coma, and foot ulcers. The condition develops due to abnormalities in carbohydrate metabolism and insulin synthesis resulting in high blood sugar with symptoms such as elevated hunger and thirst, polyuria, glycosuria, and lethargy. The World Health Organization [1] has predicted that the worldwide number of patients with diabetes will double by the year 2025, from the current number of approximately 150 million to 300 million. Studies have shown that during the manifestations of diabetes there is an enhanced production of free radicals and reactive oxygen species (ROS), which enhanced lipid peroxidation, damage to DNA, and protein degradation. In type 1 diabetes, ROS are involved in *β*-cell dysfunction initiated by autoimmune reactions and inflammatory cytokines [2]. In type 2 diabetes, ROS activate *β*-cell apoptotic pathways, impair insulin synthesis, and also contribute to insulin resistance [3, 4]. Despite the great strides made in the understanding and management of diabetes, the disease and disease related complications are increasing unabatedly due to multiple defects in its pathophysiology [5]. Many therapeutic approaches have been utilised for treatment of this disorder including the use of oral hypoglycaemic agents. The currently available oral antidiabetic agents (sulfonylureas, biguanides and meglitinides, thiazolidinediones, and *α*-glucosidase inhibitors) are used as monotherapy or in combination to achieve better glycaemic control. Metformin, a biguanide antihyperglycaemic agent, is widely used in the management of type 2 diabetes mellitus. It lowers the blood glucose concentration without causing hypoglycaemia [6]. Urakami et al. [7] reported that metformin may represent a useful adjuvant to the management of type 1 diabetes mellitus. Glibenclamide belongs to the sulfonylurea class of oral drugs that reduce blood glucose levels by stimulating insulin secretion. In the presence of viable pancreatic *β*-cells, sulfonylureas enhance the release of endogenous insulin, thereby reducing blood glucose levels. Repaglinide is a prandial glucose regulator used in the management of type 2 diabetes mellitus [8]. It belongs to the meglitinide class of short-acting insulin secretagogues, which act by binding to *β*-cells of the pancreas to stimulate insulin release. While the potential of oral hypoglycaemic drugs (especially metformin, glibenclamide, and repaglinide) in treating diabetes has well been investigated, there is only little information to support their protection against oxidative stress-induced damage during diabetic complications. In this study, we investigated the effects of metformin (MET), glibenclamide (GLI), and repaglinide (REP) in protection against oxidative stress and damage using animal model.

## 2. Methods

Twenty-four adult male albino rats (100–160 g) were obtained from the Animal House of the Faculty of Pharmaceutical Sciences, University of Nigeria, Nsukka. The animals were housed at 25 ± 2°C under 12-hour light/dark cycle maintained on a standard feed and water* ad libitum.* The rats were fasted for 12 hours with free access to water prior to the administration of freshly prepared alloxan monohydrate (130 mg/kg; i.p.) dissolved in ice-cold normal saline. After 5 days of stabilisation of diabetes, animals having fasting blood glucose concentration ≥200 mg/dL (11.1 mmol/L) were considered diabetic and used for the investigation. The animals were divided into four (4) groups (*n* = 6). Group 1 was used as the control (untreated group) while groups 2, 3, and 4 received MET (25 mg/kg, p.o.), GLI (2.5 mg/kg, p.o.), and REP (0.5 mg/kg, p.o.), respectively. All drugs were given orally once daily for fourteen (14) days. At the end of the experimental period, animals were sacrificed. Serum was obtained for further biochemical analysis. All animal experiments were conducted in compliance with the National Institute of Health Guide for Care and Use of Laboratory Animals (Pub. number 85-23, revised 1985) and in accordance with the University Ethics Committee on the use of laboratory animals.

### 2.1. Drugs/Reagents

All drugs and reagents used were obtained commercially and of analytical grade and products of May and Baker, England; BDH, England; Merck, Darmstadt, Germany; Accu-check active glucometer by Roche Diagnostic, Germany; alloxan monohydrate, Sigma-Aldrich Chemical (St. Louis, MO, USA).

### 2.2. Estimation of Biochemical Analysis

Superoxide dismutase (SOD) activity was assayed using kit product of Randox Diagnostics according to the method of Xin et al. [9]. Catalase activity was measured using kit product of Randox Diagnostics according to the method described by Aebi [10]. Reduced glutathione (GSH) was determined using the modified method of King and Wotton [11]. Malondialdehyde (MDA) concentration was determined by measuring spectrophotometrically the level of the lipid peroxidation product, malondialdehyde (MDA), as described by Varshney and Kale [12].

### 2.3. Statistical Analysis

The data obtained were analysed using Statistical Package for Social Sciences (SPSS), version 18. Results were expressed as mean ± SD (*n* = 6). The data was analysed using One-Way Analysis of Variance (ANOVA) followed by* Post Hoc* Dunnett's test. ^*∗*^ *p* < 0.05 was considered to be statistically significant.

## 3. Results

### 3.1. Effects of Metformin, Glibenclamide, and Repaglinide on Serum Superoxide Dismutase Activity

**Figure 1:**
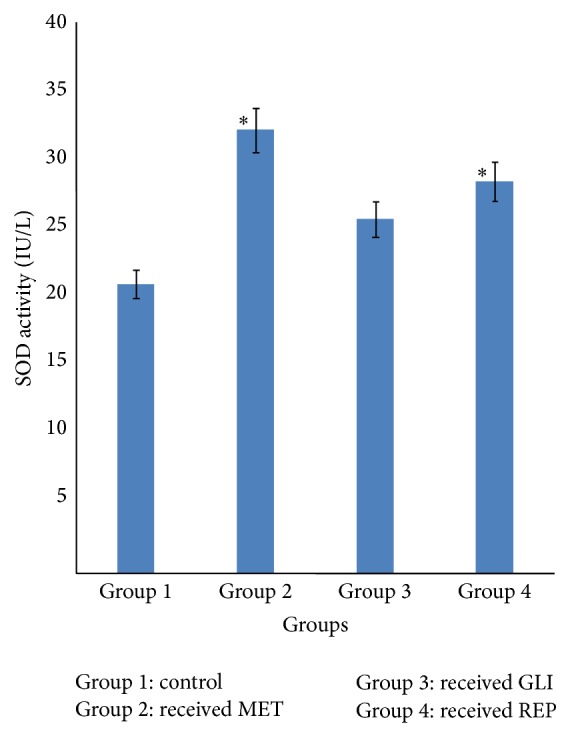
Effects of metformin (MET), glibenclamide (GLI), and repaglinide (REP) on serum superoxide dismutase activity. Data are expressed as mean ± SEM (*n* = 6), ^*∗*^ *p* < 0.05 when compared with control group.

Serum superoxide dismutase (SOD) activity of diabetic rats in groups 2 and 4 given MET (25 mg/kg) and REP (0.5 mg/kg) was found to be significantly (*p* < 0.05) higher when compared with the diabetic control rats in group 1. However, the serum SOD activity of diabetic rats in group 3 given GLI (2.5 mg/kg) was observed to be insignificantly (*p* > 0.05) higher when compared with the diabetic control (Figure 1).

### 3.2. Effects of Metformin, Glibenclamide, and Repaglinide on Serum Catalase Activity

**Figure 2:**
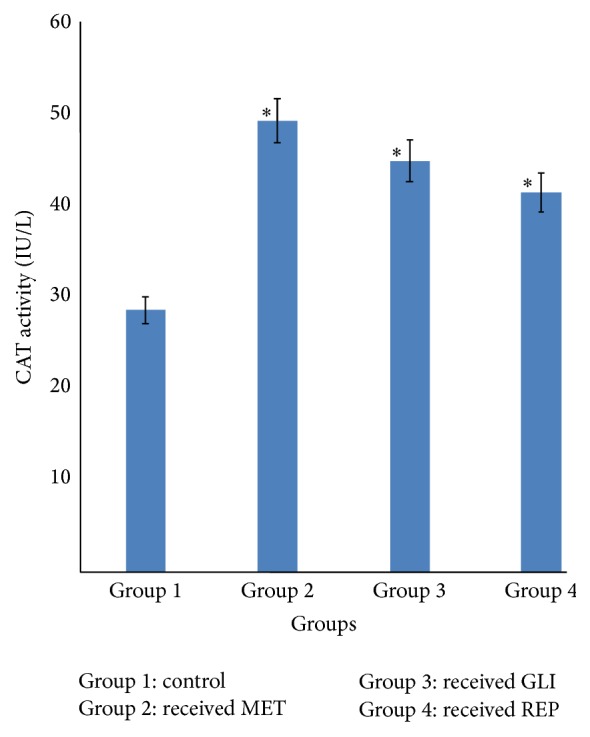
Effects of metformin (MET), glibenclamide (GLI), and repaglinide (REP) on serum catalase activity. Data are expressed as mean ± SEM (*n* = 6), ^*∗*^ *p* < 0.05 when compared with control group.

The serum catalase activity of diabetic rats in groups 2, 3, and 4 given MET (25 mg/kg), GLI (2.5 mg/kg), and REP (0.5 mg/kg), respectively, was observed to be significantly (*p* < 0.05) higher compared with the diabetic control in group 1 (Figure 2).

### 3.3. Effects of Metformin, Glibenclamide, and Repaglinide on Serum Glutathione (GSH) Concentration

**Figure 3:**
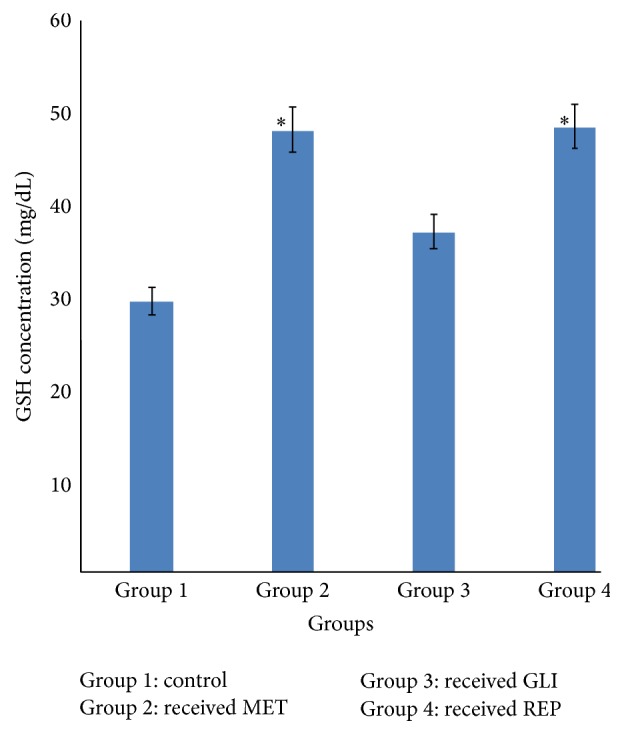
Effects of metformin (MET), glibenclamide (GLI), and repaglinide (REP) on serum glutathione (GSH) concentration. Data are expressed as mean ± SEM (*n* = 6), ^*∗*^ *p* < 0.05 when compared with control group.

The glutathione (GSH) concentration of diabetic rats in groups 2 and 4 given MET (25 mg/kg) and REP (0.5 mg/kg), respectively, was observed to be significantly (*p* < 0.05) higher compared with the diabetic control. However, the GSH concentration of diabetic rats in group 3 given GLI (2.5 mg/kg) was observed to be insignificant (*p* > 0.05) compared with the diabetic control (Figure 3).

### 3.4. Effects of Metformin, Glibenclamide, and Repaglinide on Serum Malondialdehyde Concentration

**Figure 4:**
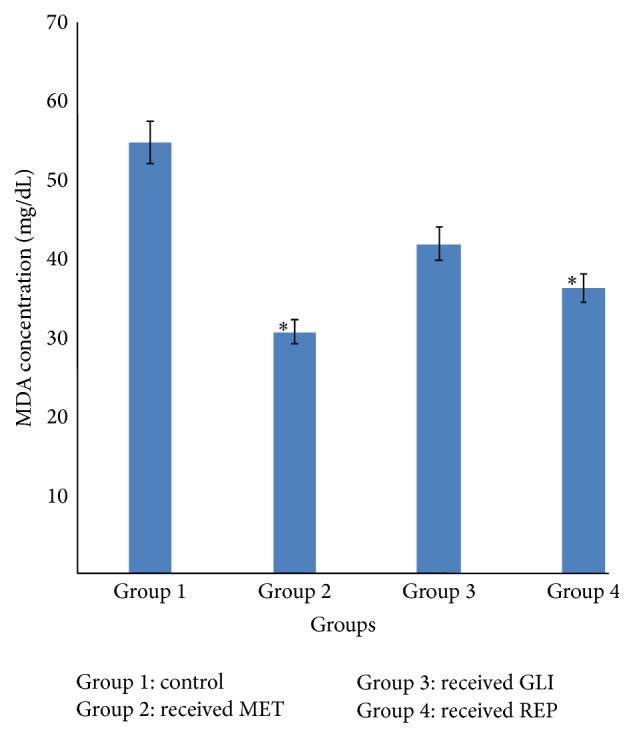
Effects of metformin (MET), glibenclamide (GLI), and repaglinide (REP) on serum malondialdehyde concentration. Data are expressed as mean ± SEM (*n* = 6), ^*∗*^ *p* < 0.05 when compared with control group.

Significantly (*p* < 0.05) lower concentrations of serum malondialdehyde (MDA) were observed in groups 2 and 4 diabetic rats given MET (25 mg/kg) and REP (0.5 mg/kg), respectively, compared with the serum MDA concentration of diabetic control rats (group 1). However, there was no significant (*p* > 0.05) reduction observed in the serum MDA concentration of diabetic rats in group 3 given GLI (2.5 mg/kg) compared with the diabetic control (Figure 4).

## 4. Discussion

The present study investigated the antioxidant effects of three standard antidiabetic agents belonging to three different classes, metformin (MET), glibenclamide (GLI), and repaglinide (REP), in alloxan-induced diabetic rats. The oxidative stress induced by alloxan arises due to a compromise in natural antioxidant mechanism and an increase in oxygen free radical production [13, 14]. Reactive oxygen species- (ROS-) induced oxidative damage has been implicated in the pathogenesis of several disorders including diabetes mellitus (DM) [15]. This may lead to imbalance of* in vivo* antioxidant status as evaluated by the activities of enzymatic and concentration of nonenzymatic (GSH) antioxidant in this study. Free radical scavenging enzymes such as superoxide dismutase (SOD) and catalase (CAT) protect the biological system from oxidative stress [16, 17]. SOD catalyses the reaction in which superoxide anion is converted to hydrogen peroxide and oxygen while catalase is a haem-containing ubiquities enzyme that detoxifies H_2_O_2_ into water and oxygen [18]. The reductions observed in the activities of SOD and CAT in the diabetic control group suggest their excessive utilisation in attenuating free radicals generated during the metabolism of alloxan. This observation has already been reported in diabetic animals [19, 20]. Increase in their activities is an indication of their ability to scavenge ROS, thus contributing to the protective effect against oxidative stress and preventing further damage to membrane lipids. The increase in SOD and CAT activity observed in MET and GLI treated diabetic rats is in agreement with previous reports [20, 21]. Nonenzymatic antioxidant (reduced glutathione) acts synergistically to scavenge the free radicals formed in the biological system, thus preventing cells from oxidative damage. GSH is an intracellular thiol rich tripeptide, which plays a major role in the balance of oxidative stress by protection of cells and tissue structures [22, 23]. In the diabetic control group, the reduced GSH level may be due to its increased utilisation during oxidative stress to scavenge free radicals. However, the reduced GSH level was reversed with the administration of MET, GLI, and REP to the diabetic rats. This may suggest that the standard antidiabetic agents maintained the levels of GSH that are utilised during diabetes and hence also stabilised lipid peroxidation.

Lipid peroxidation of unsaturated fatty acids is frequently used as an indicator of oxidative stress and subsequent oxidative damage [24, 25]. Lipid peroxidation impairs cell membrane fluidity and alters the activity of membrane-bound enzymes and receptors resulting in membrane malfunction [26]. The increased lipid peroxidation during diabetes as found in the increased concentration of malondialdehyde (MDA), an end product of lipid peroxidation in the diabetic control rats, is a reflection of enhanced oxidative damage to lipids. Similar reports have shown an elevation in the status of lipid peroxidation in the liver after alloxan induction [27–29]. This suggests that peroxidative injury may be involved in the development of diabetic complications. However, following oral administration of MET, GLI, and REP, the MDA level reduced considerably. The reduction in the MDA level observed in MET and GLI treated diabetic rats is similar to previous reports [20, 21]. However, the degree of effects of these standard antidiabetic agents was in the order of MET > REP > GLI. This may be attributed to the fact that MET, GLI, and REP may have some protective effect against lipid peroxidation, thereby contributing to the protection against oxidative damage in diabetes.

In conclusion, this study has shown that oxidative stress is still evident during diabetic manifestations. The administration of metformin (MET), glibenclamide (GLI), and repaglinide (REP) exhibited significant reduction in the malondialdehyde (MDA) concentration and considerable improvement in the altered activities of antioxidant enzymes. This establishes the fact that they provide additional antioxidant protection as antidiabetic drugs, thereby protecting the pancreas from oxidative stress-induced damage during diabetic complications.

## References

[1] World Health Organization (2002). *Diabetes Mellitus Fact Sheet No. 138*.

[2] Cnop M., Welsh N., Jonas J.-C., Jörns A., Lenzen S., Eizirik D. L. (2005). Mechanisms of pancreatic *β*-cell death in type 1 and type 2 diabetes: many differences, few similarities. *Diabetes*.

[3] Evans J. L., Goldfine I. D., Maddux B. A., Grodsky G. M. (2003). Are oxidative stress activated signaling pathways mediators of insulin resistance and *β*-cell dysfunction?. *Diabetes*.

[4] Simmons R. A. (2006). Developmental origins of diabetes: the role of oxidative stress. *Free Radical Biology and Medicine*.

[5] Ivorra M. D., Payá M., Villar A. (1989). A review of natural products and plants as potential antidiabetic drugs. *Journal of Ethnopharmacology*.

[6] Scheen A. J. (1996). Clinical pharmacokinetics of metformin. *Clinical Pharmacokinetics*.

[7] Urakami T., Morimoto S., Owada M., Harada K. (2005). Usefulness of the addition of metformin to insulin in pediatric patients with type 1 diabetes mellitus. *Pediatrics International*.

[8] Polonsky K. S., Given B. D., Hirsch L. J. (1988). Abnormal patterns of insulin secretion in non-insulin-dependent diabetes mellitus. *The New England Journal of Medicine*.

[9] Xin Z., Waterman D. F., Hemken R. W., Harmon R. J. (1991). Effects of copper status on neutrophil function, superoxide dismutase, and copper distribution in steers. *Journal of Dairy Science*.

[10] Aebi H. E. (1983). Catalase. *Methods of Enzymatic Analysis*.

[11] King E. J., Wotton I. D. P. (1959). Microanalysis. *Medical Biochemistry*.

[12] Varshney R., Kale R. K. (1990). Effects of calmodulin antagonists on radiation-induced lipid peroxidation in microsomes. *International Journal of Radiation Biology*.

[13] Baynes J. W., Thorpe S. R. (1999). Role of oxidative stress in diabetic complications. *Diabetes*.

[14] El-Demerdash F. M., Yousef M. I., El-Naga N. I. A. (2005). Biochemical study on the hypoglycemic effects of onion and garlic in alloxan-induced diabetic rats. *Food and Chemical Toxicology*.

[15] Pitocco D., Zaccardi F., Di Stasio E. (2010). Oxidative stress, nitric oxide, and diabetes. *Review of Diabetic Studies*.

[16] Comporti M. (1985). Lipid peroxidation and cellular damage in toxic liver injury. *Laboratory Investigation*.

[17] Del Rio D., Stewart A. J., Pellegrini N. (2005). A review of recent studies on malondialdehyde as toxic molecule and biological marker of oxidative stress. *Nutrition, Metabolism and Cardiovascular Diseases*.

[18] Selvan V. T., Manikandan L., Senthil Kumar G. P. (2008). Antidiabetic and antioxidant effect of methanol extract of *Artanema sesamoides* in streptatozocin-induced diabetic rats. *International Journal of Applied Research in Natural Products*.

[19] Pavana P., Sethupathy S., Santha K., Manoharan S. (2009). Effects of *Tephrosia purpurea* aqueous seed extract on blood glucose and antioxidant enzyme activities in streptozotocin induced diabetic rats. *African Journal of Traditional, Complementary and Alternative Medicines*.

[20] Onyeka C. A., Nwakanma A. A., Bakare A. A. (2013). Hypoglycemic, antioxidant and hepatoprotective activities of ethanolic root bark extract of *Chrysophyllum albidum* in alloxan-induced diabetic rats. *Bangladesh Journal of Medical Science*.

[21] Amarnath Satheesh M., Pari L. (2004). Antioxidant effect of *Boerhavia diffusa* L. in tissues of alloxan-induced diabetic rats. *Indian Journal of Experimental Biology*.

[22] Yu B. P. (1994). Cellular defenses against damage from reactive oxygen species. *Physiological Reviews*.

[23] Ahmed M. M., Ahmed A. E., Hala S. A. G., Gehan M. M., Fahad A. A. (2011). Protective effects of simvastatin, an HMG-CoA reductase inhibitor, against oxidative damage in experimental diabetic rats. *International Journal of PharmTech Research*.

[24] Haugaard N. (1968). Cellular mechanisms of oxygen toxicity. *Physiological Reviews*.

[25] Erejuwa O. O., Sulaiman S. A., Abdul Wahab M. S., Salam S. K. N., Salleh M. S. M., Gurtu S. (2010). Antioxidant protective effect of glibenclamide and metformin in combination with honey in pancreas of streptozotocin-induced diabetic rats. *International Journal of Molecular Sciences*.

[26] Halliwell B. (2000). Lipid peroxidation, antioxidants and cardiovascular disease: how should we move forward?. *Cardiovascular Research*.

[27] Rauscher F. M., Sanders R. A., Watkins J. B. (2000). Effects of new antioxidant compounds PNU-104067F and PNU-74389G on antioxidant defense in normal and diabetic rats. *Journal of Biochemical and Molecular Toxicology*.

[28] Zheng W., Wang S. Y. (2001). Antioxidant activity and phenolic compounds in selected herbs. *Journal of Agricultural and Food Chemistry*.

[29] Iweala E. E. J., Okeke C. U. (2005). Comparative study of the hypoglycemic and biochemical effects of *Catharanthus roseus* (Linn) G. apocynaceae (Madagascar periwinkle) and chlorpropamide (diabinese) on alloxan-induced diabetic rats. *Biokemistri*.

